# Supplementary material for: Design, Preparation, Characterization and Evaluation of Five Cocrystal Hydrates of Fluconazole with Hydroxybenzoic Acids
Source: Pharmaceutics. 2022 Nov 17;14(11):2486. doi: 10.3390/pharmaceutics14112486 (PMC9694130; doi:10.3390/pharmaceutics14112486)
Supplement: Supplementary file 1 [file pharmaceutics-14-02486-s001.zip › pharmaceutics-2020464-supplementary.pdf]

Design, Preparation, Characterization and Evaluation of Five Cocrystal Hydrates of Fluconazole with Hydroxybenzoic Acids

Hongmei Yu[a], Baoxi Zhang[a], Meiju Liu[a],Wenhui Xing[a], Kun Hu[a], Shiying Yang[a], Guorong He[b], Ningbo Gong[a,*], Guanhua Du[b], Yang Lu[a,* *].

[a] *Beijing Key Laboratory of Polymorphic Drugs, Institute of Materia Medica, Chinese Academy of Medical Sciences and Peking Union Medical College, Beijing 100050, China*

[b] *Beijing City Key Laboratory of Drug Target Identification and Drug Screening, Institute of Materia Medica, Chinese Academy of Medical Sciences and Peking Union Medical College. Beijing 100050, China.*

Table S1

Hydrogen Bond Geometrical Parameters of Crystal Structures.

compound	interation	H···A/ Å	D···A/ Å	∠(DHA)/ °	Symmetry
FLZ–24DHB–H_2O	$O_1–H_1\cdots O_6$	1.922	2.735	171.31	intramolecular
	$O_{2^a}–H_{2A^a}\cdots N_3$	2.017	2.722	143.81	intramolecular
(1:0.5:1)	$O_{5^a}–H_{5^a}\cdots N_3$	1.776	2.595	176.04	[-x+1, -y, -z+2]
FLZ–34DHB–H_2O	$O_1–H_1\cdots O_6$	1.919	2.729	169.09	intramolecular
(1:0.5:1)	$O_{3^a}–H_{3^a}\cdots N_3$	1.862	2.673	169.48	intramolecular
	$O_1–H_1\cdots O_2$	2.217	2.975	153.94	[-x, y+1/2, -z+1]
FLZ–34DHB–H_2O	$O_3–H_3\cdots O_6$	1.777	2.596	176.11	[-x+1, y-1/2, -z+1]
	$O_4–H_{4A}\cdots N_3$	2.021	2.778	153.32	[-x, y+1/2, -z+1]
(1:1:1)	$O_4–H_{4A}\cdots O_5$	2.292	2.725	113.53	intramolecular
	$O_5–H_{5A}\cdots N_6$	1.930	2.708	157.91	[x-1, y, z]
	$O_1–H_1\cdots O_6$	1.905	2.716	170.09	intramolecular
FLZ–35DHB–H_2O	$O_2–H_{2A}\cdots N_6$	1.916	2.691	157.28	intramolecular
(1:1:1)	$O_3–H_3\cdots N_3$	1.922	2.727	166.98	[-x+3/2, y+1/2, -z+1/2]
	$O_4–H_{4A}\cdots N_5$	1.917	2.736	177.61	[x+1, y, z+1]
	$O_1–H_1\cdots O_7$	2.059	2.832	157.04	[-x+1, y+1/2, -z+3/2]
	$O_2–H_{2A}\cdots N_6$	1.786	2.689	177.45	intramolecular
FLZ–345THB–H_2O	$O_4–H_{4A}\cdots O_2$	2.121	2.873	152.35	[x, -y+3/2, z-1/2]
(1:1:1)	$O_5–H_{5A}\cdots N_3$	1.991	2.771	158.80	[-x+1, y+1/2, -z+3/2]
	$O_5–H_{5A}\cdots O_6$	2.328	2.750	112.58	intramolecular
	$O_6–H_{6A}\cdots O_7$	1.934	2.752	174.94	intramolecular

* Corresponding author.
** Corresponding author.
E-mail addresses: gnb@imm.ac.cn (N.B. Gong), luy@imm.ac.cn (Y. Lu).

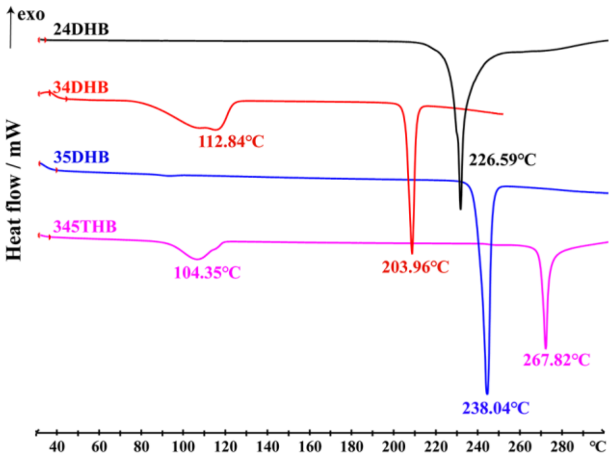

Figure S1. DSC thermograms of CCFs used in the study.

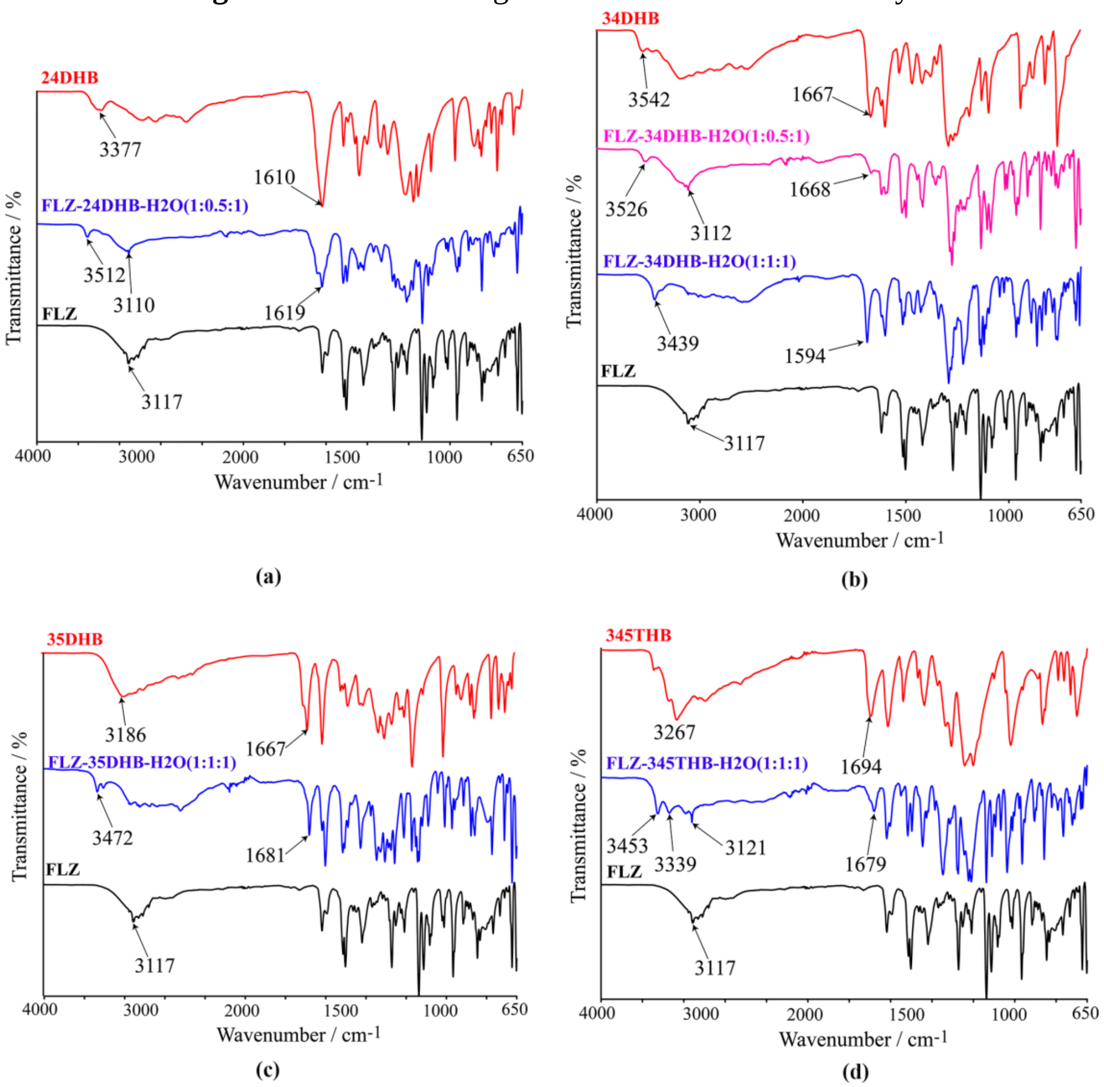

Figure S2. FT−IR spectra of starting components, and synthesized cocrystals (a) FLZ−24DHB−H_2O (1:0.5:1) , (b) FLZ−34DHB−H_2O (1:1:1), FLZ−34DHB−H_2O (1:0.5:1), (c) FLZ−35DHB−H_2O (1:1:1), (d) FLZ−345THB−H_2O (1:1:1).

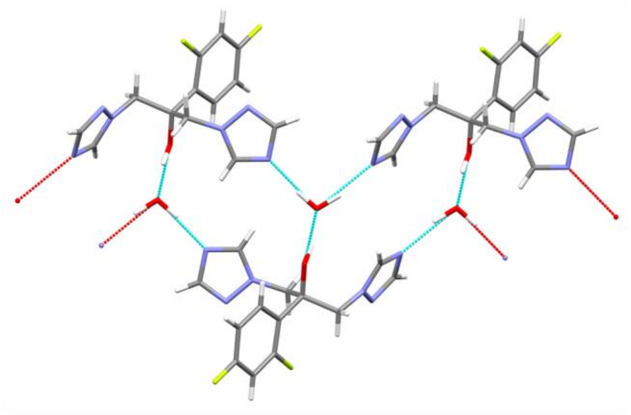

Figure S3. Hydrogen bond interactions between FLZ and water molecules in the form of FLZ monohydrate (CSD refcode IVUQIZ).
